# Radixin inhibition decreases adult neural progenitor cell migration and proliferation in vitro and in vivo

**DOI:** 10.3389/fncel.2013.00161

**Published:** 2013-09-24

**Authors:** Åsa Persson, Olle R. Lindberg, Hans G. Kuhn

**Affiliations:** Center for Brain Repair and Rehabilitation, Institute of Neuroscience and Physiology, Sahlgrenska Academy, University of GothenburgGothenburg, Sweden

**Keywords:** ezrin/radixin/moesin, subventricular zone, rostral migratory stream, neuronal migration, proliferation

## Abstract

Neuronal progenitors capable of long distance migration are produced throughout life in the subventricular zone (SVZ). Migration from the SVZ is carried out along a well-defined pathway called the rostral migratory stream (RMS). Our recent finding of the specific expression of the cytoskeleton linker protein radixin in neuroblasts suggests a functional role for radixin in RMS migration. The ezrin-radixin-moesin (ERM) family of proteins is capable of regulating migration through interaction with the actin cytoskeleton and transmembrane proteins. The ERM proteins are differentially expressed in the RMS with radixin and moesin localized to neuroblasts, and ezrin expression confined to astrocytes of the glial tubes. Here, we inhibited radixin function using the quinocarmycin analog DX52-1 which resulted in reduced neuroblast migration *in vitro,* while glial migration remained unaltered. Furthermore, the morphology of neuroblasts was distorted resulting in a rounded shape with no or short polysialylated neural cell adhesion molecule positive processes. Intracerebroventricular infusion of the radixin inhibitor resulted in accumulation of neuroblasts in the anterior SVZ. Neuroblast chains were short and intermittently interrupted in the SVZ and considerably disorganized in the RMS. Moreover, we studied the proliferation activity in the RMS after radixin inhibition, since concentrated radixin expression has been demonstrated in the cleavage furrow of dividing cells, which indicates a role of radixin in cell division. Radixin inhibition decreased neuroblast proliferation, whereas the proliferation of other cells in the RMS was not affected. Our results demonstrate a significant role for radixin in neuroblast proliferation and migration.

## INTRODUCTION

In the adult rodent brain neuronal progenitor cells, neuroblasts, migrate a long distance from the neurogenic subventricular zone (SVZ) through the rostral migratory stream (RMS) to their final destination in the olfactory bulb (OB) where they differentiate into mature neurons (Altman, 1969; Lois and Alvarez-Buylla, 1994). The significance and extent of adult neurogenesis in the adult human SVZ/RMS area is not clear, however, in rodents brain injury and pathology can induce both proliferation and deviated migration from the SVZ/RMS toward damaged tissue areas (Arvidsson et al., 2002; Li et al., 2010; Osman et al., 2011). Abolishing migrating neuroblasts after stroke leads to worsened recovery (Jin et al., 2010), suggesting a supportive role for these neuronal progenitors under injury conditions. A deeper understanding of RMS migration may reveal ways to direct immature cells to damaged areas and to increase possibilities for brain repair. In the RMS, neuroblasts migrate along each other forming cellular chains which are tightly surrounded by glial cells, often referred to as the glial tubes (Lois et al., 1996; Peretto et al., 1997). Chain migration has been proposed to be supported by the glial tubes and blood vessels and, is regulated by numerous extracellular and intracellular cues. Polysialylated neural cell adhesion molecule (PSA-NCAM) is important for the organization of chain migration in the RMS and adjusts cell-cell adhesion (Ono et al., 1994; Chazal et al., 2000). Doublecortin (DCX) is required for nuclear translocation during neuroblast migration (Gleeson et al., 1999; Koizumi et al., 2006). Moreover, neuroblasts divide en route to the OB and EGF receptors (Anton et al., 2004; Kim et al., 2009); ephrins (Conover et al., 2000; Ghashghaei et al., 2006) and NogoA (Rolando et al., 2012) have been found to regulate both migration and proliferation in the RMS. Cellular functions such as migration and adhesion require a highly dynamic cytoskeleton. Linker proteins of the ERM (ezrin/radixin/moesin) family can interact with both f-actin and several transmembrane proteins, providing a connection between extracellular cues and the cytoskeleton (Sato et al., 1992). The action of ERM proteins is regulated by binding of their two main domains; the N-terminal FERM domain and the C-terminal domain, which can be interrupted by threonine phosphorylation (for review, see Bretscher et al., 2002). An open conformation enables simultaneous binding of ERM proteins to the cytoskeleton and to transmembrane proteins such as receptors, ECM molecules and adhesion proteins (Serrador et al., 1997; Ivetic et al., 2002; Bono et al., 2005; Loebrich et al., 2006; Takai et al., 2007; Tang et al., 2007; Terawaki et al., 2008). Formation of actin-rich structures like filopodia and lamellipodia are essential for cell migration and process formation in various cell types. The involvement of ERM proteins in a variety of cell functions in the embryonic and early postnatal brain, including axonal outgrowth, morphological rearrangement, cell migration and signaling, have been demonstrated (Paglini et al., 1998; Castelo and Jay, 1999; Loebrich et al., 2006; Haas et al., 2007; Parisiadou et al., 2009). In the adult brain, however, ERM proteins have been less studied. Although highly homologous, the ERM proteins seem to localize to different cell types in the adult brain, with ezrin expression in glial cells and radixin expression in neuronal cells (Paglini et al., 1998; Gronholm et al., 2005; Cleary et al., 2006; Persson et al., 2010). We recently described the specific expression of radixin in PSA-NCAM^+^ neuroblasts in the adult SVZ and RMS (Persson et al., 2010). Here we investigate the function of radixin in neuroblasts using the radixin inhibitor DX52-1, aquinocarmycin analog. The inhibitor was recently shown to primarily target radixin and disrupt the ability for radixin to bind actin as well as transmembrane proteins, such as CD44 (Kahsai et al., 2006). In this study, we explore the effects of DX52-1 and radixin inhibition on neuroblasts in the adult SVZ and RMS using *in vitro* and *in vivo* approaches to analyze migration, proliferation, cell death, and proteomic changes.

## MATERIALS AND METHODS

### CHEMICALS

The quinocarmycin analog DX52-1 (generous gift from Prof. Gabriel Fenteany) was used to block radixin function as described previously (Kahsai et al., 2006). A stock solution was prepared by dissolving the compound in sterile 50% DMSO in PBS which was further diluted with PBS to the final concentration (<0.05% for *in vivo* experiments and <0.0025% DMSO for *in vitro* experiments). Control experiments were always performed with the same concentration of DMSO as the corresponding diluted DX52-1 solution.

### ANIMALS

Eight to nine week-old male Wistar rats were used in this study. All rats were housed in a barrier facility with a 12-h light/dark cycle and allowed free access to food and water. Experiments were conducted according to protocols approved by the Gothenburg ethics committee of the Swedish Animal Welfare Agency (Ethical application no 32/11 and 145/10). For *in vitro* studies and whole mount preparations, animals were anesthetized using isofluorane and brains removed after decapitation.

### SURGERY

Surgeries were performed under ketamine (33 mg/mL Ketalar, Pfizer, New York, NY, USA) and xylazine (6.67 mg/mL Rompun, Bayer Healthcare AG, Tarrytown, NY, USA) anesthesia, and all efforts were made to minimize suffering. The animals were divided into two groups receiving either vehicle (0.05% DMSO in PBS) or DX52-1 (1.3 μg/day), for 4 days. The surgeries were performed as previously described (Lindberg et al., 2012). Briefly, osmotic minipumps (Model 1002; Alzet-Durect, Cupertino, CA, USA) and infusion cannulas (Brain Infusion Kit 2; Alzet-Durect) were filled with vehicle or DX52-1. Cannulas were inserted intracerebroventricularly using a stereotaxic instrument [David Kopf, Tujunga, CA and Stoelting Co, Wood Dale, IL, USA; anteroposterior (AP) +8.5 mm, lateral +1.2 mm from the center of the interaural line at flat skull position; cannula length, 5 mm below skull] and the minipumps were placed subcutaneously. At the end of the DX52-1 infusion period, animals were sedated using an overdose of pentobarbital and transcardially perfused with 4% PFA (Histolab, Gothenburg, Sweden) in 0.1 M phosphate buffer (pH 7.4). Brains were removed, postfixed for 24 h in 4% PFA (Thermo Fisher Scientific, Waltham, MA, USA) and thereafter kept in 30% sucrose at 4°C until further processed.

### IMMUNOFLUORESCENCE

The ipsilateral side of infused brains was cut in a sagittal plane and the contralateral side was cut coronally. Sagittal sections were cut at 25 μm and coronal sections were cut at 40 μm on a sliding microtome (Leica Microsystems, Wetzlar, Germany) followed by immunofluorescence. Immunostainings including radixin were preceded by antigen retrieval in sodium citrate, pH 6.0, for 20 min at 97°C followed by 15 min cooling at room temperature. Sections were blocked for 30 min in 3% normal donkey serum (Jackson ImmunoResearch, West Grove, PA, USA) in 0.1% Triton X-100, and then incubated for 48 h at 4°C in primary antibodies; monoclonal rabbit anti-radixin (Abcam, Cambridge, MA, USA), mouse anti-radixin (Abnova, Taipei City, Taiwan), rabbit anti-phosphorylated ezrin/radixin/moesin (Cell signaling, Danvers, MA, USA), rabbit anti-phosphorylated histone H3 (PHH3, Millipore, Billerica, MA, USA), mouse IgM anti-PSA-NCAM (Chemicon International/Millipore, Billerica, MA, USA). ToPro-3 (Molecular Probes/Invitrogen, Carlsbad, CA, USA) was used as a nuclear counterstain. After rinsing in tris-buffered saline (TBS), sections were incubated for 2 h with Alexa Fluor-conjugated secondary antibodies (Molecular Probes) and CF secondary antibodies (Biotium, Hayward, CA, USA). The sections were mounted on glass slides and coverslipped with ProLong Gold DAPI (Molecular Probes).

To study apoptotic cell death in the SVZ and RMS after DX52-1 infusion, the ApopTag Fluorescein Direct in situ Apoptosis Detection kit (Millipore) was used. Fixed free floating sections were mounted onto glass slides and pretreated with ethanol:acetic acid (2:1) for 5 min at -20°C followed by a PBS washing step. After 1 h of incubation in terminal deoxynucleotidyltransferase at 37°C, the reaction was stopped by washing and the sections were incubated for 30 min with a Fluorescein-conjugated anti-digoxigenin antibody at room temperature and subsequently washed in PBS. The slides were coverslipped with ProLong Gold DAPI (Molecular Probes).

### SVZ WHOLE MOUNT PREPARATION

After 4 days of vehicle or DX52-1 intracerebroventricular infusion, brains (*n* = 3) were removed and placed in 37°C warm Hank’s Balansed Salt Solution (HBSS, Invitrogen). The whole ventricular wall of the contralateral hemisphere, including the underlying parenchyma, was carefully dissected out and fixed in cold 4% PFA/0.1% Triton X-100 in PBS for 24 h before washing and blocking unspecific binding in 10% Donkey serum/2% Triton X-100 in PBS for 1 h (Mirzadeh et al., 2010). The wholemount was incubated for 48 h with primary antibodies; goat anti-DCX (Santa Cruz Biotechnology, Inc., Santa Cruz, CA, USA), rabbit anti-PhosH3 (Millipore), washed thoroughly in 0.1% Triton X-100 in PBS, and subsequently incubated for 24 h in Alexa Fluor secondary antibodies (Molecular Probes). After completing the staining, a sliver of the SVZ was cut out from the underlying parenchyma and coverslipped with Prolong Gold DAPI (Molecular Probes).

### SVZ EXPLANTS

For explant cultures brains were rapidly removed and kept in Hank’s balanced salt solution (Gibco/Invitrogen) on ice. One millimeter coronal brain slices were cut between anterio-posterior coordinates Bregma -0.5 to 2.5 using a coronal brain matrix. The slices were kept on ice while the lateral ventricle walls were dissected and cut into approximately 100 μm diameter pieces. The tissue pieces were resuspended in Neurobasal A medium (Invitrogen), mixed 3:1 with Matrigel (BD Biosciences, San Jose, CA, USA) and dispensed in 8-well chamber slides (BD Bioscience), followed by 10 min polymerization at 37°C. Explants were cultured in Neurobasal A medium, supplemented with B27 and Glutamax, PenStrep (all Invitrogen) and a concentration series of the radixin inhibitor DX52-1 (Vehicle 0.0025% DMSO, 50, 100 or 250 nM) at 37°C in 5% O_2_ and 1% CO_2_ for 72 h. At the end of the experiment the explants were fixed in 4% PFA for 20 min. After three 15-min washes in TBS, the explants were blocked for 3 h at room temperature using 3% donkey serum and 0.2% Triton-X in TBS. Explants were then incubated with primary antibodies for 48 h; goat anti-Sox2 (Santa Cruz Biotechnology), rabbit anti-GFAP (DakoCytomation, Glostrup, Denmark), mouse IgM anti-PSA-NCAM (Chemicon International) and with secondary antibodies as described above.

Apoptosis and cell death was analyzed using Vybrants Apoptosis Assay kit 2 (Molecular Probes) on explants after 96 h in culture. Explants were washed in cold PBS for 10 min and subsequently in Annexin buffer for 15 min followed by incubation with Annexin V conjugated with Alexa 488 and propidium iodide (PI) in room temperature for 40 min. The cultures were washed in Annexin buffer, fixed with 2% PFA and stained with the nuclear stain ToPro-3 (Molecular Probes) before one wash in Annexin buffer and subsequently coverslipped with ProLong Gold (Molecular Probes).

### CONFOCAL MICROSCOPY AND QUANTIFICATIONS

Immunofluorescence labeling was imaged using confocal laser scanning microscope (Leica TCS SP2, Leica Microsystems, Wetzlar, Germany, and at the Centre for Cellular Imaging; Zeiss LSM 700 and Zeiss LSM 710, Carl Zeiss Microscopy GmbH, Jena, Germany). For SVZ explant cultures, migration distance of migratory chains was measured for PSA-NCAM^+^ cells. The three longest migratory chains per explant were used to estimate the maximum migration distance under DX52-1 treatment (1–4 explants). Furthermore, the percentage of PSA-NCAM^+^ and Sox2^high^ cells leaving the explants were quantified by counting the ratio of PSA-NCAM^+^ and Sox2^high^ cells (total cells counted per condition: 549 ± 66) in migratory chains emerging from the explants. Cells were visualized with the nuclear stain ToPro-3 and *n* = 4 for all explant quantifications.

For *in vivo* quantifications coronal sections from the contralateral hemisphere were used after vehicle or DX52-1 infusion. For quantification of cell proliferation in the RMS, the total number of PHH3^+^ cells, and PSA-NCAM^+^/PHH3^+^ double labeled cells, in the RMS was acquired from 7 to 9 sections at a 1:12 interval covering the RMS. Anterior RMS refers to anterio-posterior coordinates from 13.20 to 11.52 mm from interaural line and posterior RMS refers to anterio-posterior coordinates from 11.52 to 10.44 mm from interaural line. Double labeling was assumed when cells exhibited direct co-localization or when nucleus and cytosol or processes from the same cell were individually labeled. Area/volume measurements and the number of ApopTag stained cells were assessed using stereology software (Stereo Investigator; MicroBrightField Inc., Williston, VT, USA).

### PROTEOMIC ANALYSIS

The proteomic analysis was performed by the Proteomics core facility at the University of Gothenburg. Relative protein expression levels was analyzed after radixin inhibition using the TMT isotopic mass tagging kit (Thermo Fisher Scientific), where the reporter mass is used for semi quantitative identification of proteins with tandem mass spectrometry. The ipsilateral SVZ was microdissected from rats (*n* = 3) after intracerebroventricular infusion of DX52-1 or vehicle. Tissue samples were lysed in a buffer containing; 50 mM TEAB, 8 M Urea, 4% Chaps, 0.2% SDS, 5 mM EDTA, pH 8.5. Total protein concentration was determined using Pierce 660 nm Protein Assay (Thermo Fisher Scientific). 100 μg protein per sample were incubated with TCEP (tris(2-carboxyethyl)phosphine), alkylated with MMTS (methyl methanethiosulfonate) and digested with trypsin, after a four-fold dilution, in 0.5 M TEAB ratio 1:25 over night in 37°C.

TMT 6-plex reagents (126–131) were dissolved in ACN and added to the respectively sample according to manufacturer’s protocol (Thermo Fisher Scientific). After labeling and quenching of the reagents, the samples were combined and concentrated. TMT-labeled peptides were separated with strong cation exchange chromatography (SCX). The 18 peptide containing fractions were desalted on PepClean C18 spin columns according to manufacturer’s instructions (Thermo Fisher Scientific). The desalted and dried fractions were reconstituted into 0.1% formic acid and analyzed on a LTQ-Orbitrap-Velos (Thermo Fisher Scientific) interfaced with an in-house constructed nano-LC column. Two-micro liter sample injections were made with an Easy-nLCautosampler (Thermo Fisher Scientific), running at 200 nL/min. The peptides were trapped on a precolumn (45 × 0.075 mm i.d.) and separated on a reversed phase column, 200 × 0.075 mm, packed in-house with 3 μm Reprosil-Pur C18-AQ particles. The gradient was as followed; 0–60 min 5–25% acetonitrile (ACN), 0.1% formic acid, 60–75 min 25–80% ACN, 0.1% formic acid and the last 15 min at 90% ACN, 0.1% formic acid. LTQ-OrbitrapVelos settings were: spray voltage 1.4 kV, 1 microscan for MS1 scans at 60,000 resolutions (m/z 400), full mass spectrometry (MS) mass range m/z 400–1,800. The LTQ-OrbitrapVelos was operated in a data-dependent mode with one MS1 FTMS scan precursor ions followed by HCD (high energy collision dissociation), MS2 scans of the 10 most abundant protonated ions in each FTMS scan. Dynamic exclusion of a precursor during 30 s was used after one repeat for MS2. All fractions were analyzed a second time using an exclusion list of m/z for all identified peptides.

Mass spectrometry raw data files from all SCX fractions for the TMT set were merged for relative quantification and identification using Proteome Discoverer version 1.3 (Thermo Fisher Scientific). Database search was performed by Mascot search engine using the following critera: Swissprot rat protein database, MS peptide tolerance as 10 ppm, MS/MS tolerance as 0.1 Da, trypsin digestion allowing one missed cleavages with variable modifications; methionine oxidation, cysteine methylthiol, and fixed modifications; N-terminal TMT-label, lysine TMT-label. The detected protein threshold in the software was set to 99% confidence and identified proteins were grouped by sharing the same sequences to minimize redundancy.

For TMT quantification, the ratios of TMT reporter ion intensities in MS/MS spectra (m/z 126.12, 127.13, 128.13, 129.14, 130.14, 131.14) from raw data sets were used to calculate fold changes between samples. The average of all three reporters for the control group were used as the denominator. Only peptides unique for a given protein were considered for relative quantitation, excluding those common to other isoforms or proteins of the same family. The resulting ratios were then exported into Excel for data interpretation. The total group of 32 significantly changed proteins were analyzed using the software Ingenuity Pathway Analysis (IPA; Ingenuity systems, Redwood City, CA, USA).

### STATISTICS

Statistical differences of *in vivo* quantifications were analyzed using the 2-tailed Student’s *t*-test. For *in vitro* experiments, one-way ANOVA and Bonferroni post hoc test were employed. All error bars represent standard error of the mean (s.e.m.). All statistical calculations and graphical visualizations, except for the proteomics analysis, were performed in GraphPad Prism 5 (GraphPad Software, La Jolla, CA, USA). For the proteomic analysis Welsh *t*-test was used. Differences of *p* < 0.05 were considered statistically significant (*).

## RESULTS

### SPECIFIC TARGETING OF PSA-NCAM^+^ PROGENITOR CELL MIGRATION BY RADIXIN INHIBITION IN VITRO

**FIGURE 1:**
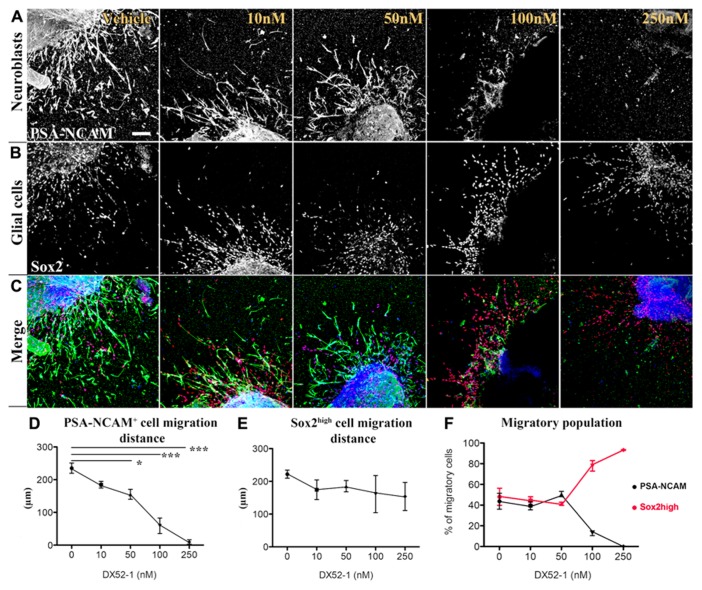
**Dose-dependent decrease in migration distance of neuronal progenitor cells from SVZ explants under radixin inhibition.** Neural and glial progenitor cells are visualized by PSA-NCAM and Sox2 immunofluorescence. **(A)** Color separation for PSA-NCAM (top row) and **(B)** Sox-2 (middle row) expressing cells under increasing concentrations of the radixin inhibitor DX52-1 (Vehicle, 10, 50, 100, and 250 nM). **(C)** Bottom row depicts color merge of PSA-NCAM (green), Sox-2 (red) immunoreactivity with Topro-3 as a nuclear stain (blue). **(D)** Migration distance (μm) of PSA-NCAM^+^ cells under DX52-1 treatment. **(E)** Migration distance (μm) of Sox2^high^ glial cells under DX52-1 treatment. **(F)** The fraction of cells expressing PSA-NCAM^+^ (black line) and Sox2^high^ (red line) in the population that migrated from the explants (*p < 0.05, **p < 0.01, ***p < 0.001). Scale bar = 100 μm.

**FIGURE 2:**
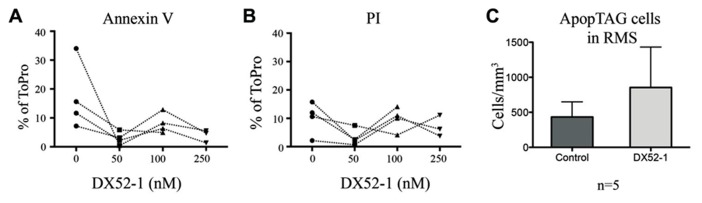
**No increase in cell death of progenitor cells *in vitro* or *in vivo* under radixin inhibition.** Paired results from four different experiments showed no significant difference in thepercentage of cells that migrated out from SVZ explant cultures being AnnexinV postitive **(A)** or PI positive **(B)** under different concentrations of the radixin inhibitor DX52-1 (Vehicle, 50, 100, and 250 nM). **(C)** The number of TUNEL positive cells was quantified per volume in the RMS after 4 days of intracerebroventricular infusion of vehicle or DX52-1 (*n* = 5).

To determine the influence of radixin inhibition on progenitor cell migration, we incubated SVZ explants with increasing concentrations of the radixin inhibitor DX52-1 for 72 h to determine the migration distance (**Figures 1A–C**). For PSA-NCAM positive neuronal progenitor cells (PSA-NCAM^+^), the average distance migrated was significantly reduced by inhibitor concentrations of 50 nM and higher (**Figure 1D**). Moreover, a reduced portion of PSA-NCAM^+^ cells migrated out from the explants under radixin inhibition at 100 and 250 nM DX52-1 (**Figure 1F**). The neuroblast chains had an altered morphology with a short leading process expressing PSA-NCAM and occasionally neuroblasts had a completely circular cell membrane and were situated close to the explant. Since a reduced migratory response could be due to toxicity of the radixin inhibitor, we assayed apoptosis and cell death using Annexin V and PI staining of cells emerging from the explants. There was no difference in the ratio of Annexin V (**Figure 2A**) or PI (**Figure 2B**) labeled cells under control conditions compared to 50, 100 or 250 nMof the inhibitor.

**FIGURE 3:**
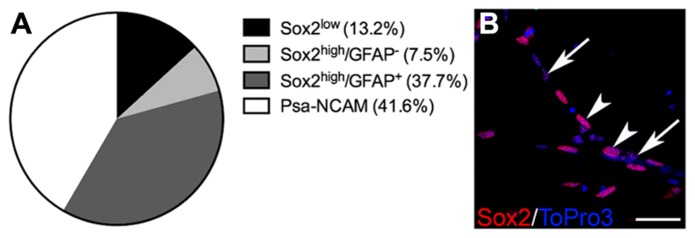
**Cell populations migrating in SVZ explant cultures. (A)** Percentage of cells positive for the markers PSA-NCAM, Sox2 and GFAP, in the SVZ explant experiment. All analyzed cells migrating out from the explants expressed Sox2, in low or high levels. Similar proportions of neuronal (PSA-NCAM^+^) and glial (GFAP^+^) cells migrated in the SVZ explants cultures and the majority of the GFAP^+^ cells expressed Sox2^high^. **(B)**_ Examples of Sox2^low^ (arrows) and Sox2^high^ (arrowheads) cells. Scale bar in **(B)** = 20 μm.

To test ifDX52-1 treatment exclusively affects migration of neuroblasts, we analyzed the migration pattern of cells expressing high levels of Sox2 (Sox2^high^), which represent the glial cells migrating from SVZ explants under control conditions. Sox2 expression is present in migrating neuroblasts, but at lower levels (**Figure 3B**) (Ferri et al., 2004). A common marker for glial cells is the glial fibrillary acidic protein (GFAP). The majority of cells migrating in SVZ explant cultures under control conditions express Sox2^high^ and a smaller fraction express GFAP (**Figure 3A**). Almost all GFAP^+^ cells were also Sox2^high^ (**Figure 3A**). No statistical difference in the migration distance of glial Sox2^high^ cells could be discerned at any concentration of the inhibitor (**Figure 1E**). Sox2^high^ cells migrated also under treatment with the highest concentration (250 nM) of the inhibitor. As a consequence, at the highest concentrations of DX52-1, the reduced migration of PSA-NCAM^+^ cells, led to an increased percentage of migratory Sox2^high^ cells surrounding the explants (**Figure 1F**).

### RADIXIN INHIBITION IN VIVO RESULTS IN DISORGANIZED NEUROBLAST CHAIN FORMATION AND ACCUMULATION IN THE POSTERIOR RMS

**FIGURE 4:**
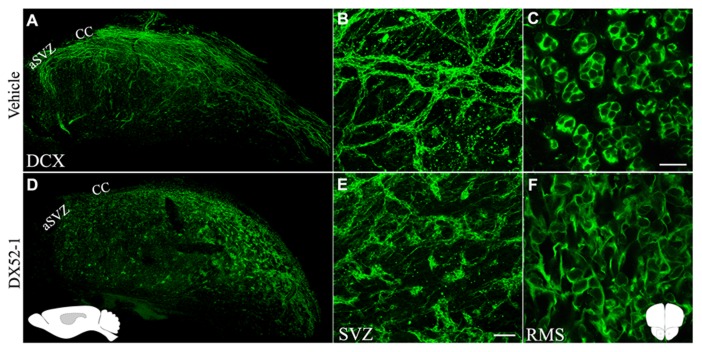
**Aberrant morphology of neuroblastsin the SVZ and RMS after radixin inhibition.** Overview **(A)** and magnification **(B)** of whole mount preparations of the lateral ventricular wall stained for DCX in control animals showing long and well organized neuroblast chains. **(C)** The RMS in a coronal section showing the tight association of DCX ^+^ neuroblasts within cellular chains in control animals. Overview **(D)** and magnification **(E)** of whole mount preparations of the lateral ventricular wall stained for DCX after DX52-1 infusion showing short and abnormalneuroblasts in aggregations. **(F)** The RMS in a coronal section showing the disorganized arrangement of DCX^+^ neuroblasts after DX52-1 infusion. Schematic inset in **(D)** represent the sagittal brain orientation, and in gray the area, of images **(A)** and **(D)**. Schematic inset in **(F)** represent the coronal orientation of images **(C)** and **(F)**. Scale bar in **(C)** = 50 μm, and **(E)** = 200 μm.

**FIGURE 5:**
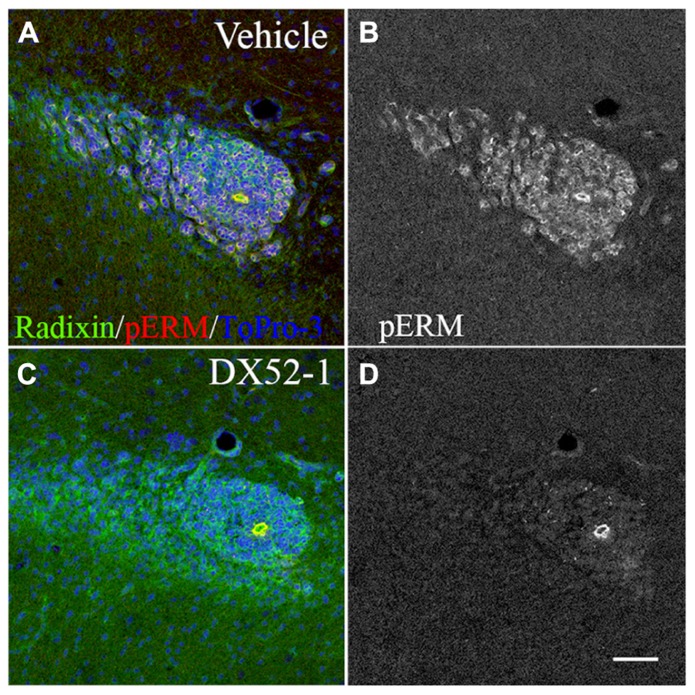
**Decreased phosphorylation of radixin in the RMS after DX52-1 treatment. (A)** Merged image of radixin and phosphorylated rzrin/radixin/moesin (pERM) immunoreactivity in a coronal section of the control RMS. **(B)** pERM immunoreactivity in the control RMS. **(C)** Merged image of radixin and phosphorylated ezrin/radixin/moesin (pERM) immunoreactivity in a coronal section in the DX52-1 treated RMS. **(D)** pERMimmunoreactivity in the DX52-1 treated RMS. Radixin/pERM immunopositive circular structures in B and D are likely residual ependymal cells originating from the wall of the collapsed olfactory ventricle (Peretto et al., 1997). Ependymal cells are known to express high levels of ezrin which explains sustained pERM expression in this area in both control and DX52-1 treated RMS. Scale bar = 50 μm.

Effects of radixin inhibitonin the SVZ and RMS were analyzed by intracerebroventricular infusion of DX52-1. Wholemount preparations of the lateral ventricle wall was used to analyze the overall organization of neuroblast chains in the SVZ. In the dorsal SVZ of naive animals, large amounts of DCX positive neuroblast chains were organized parallel to the corpus callosum and directed towards the anterior SVZ. In addition, long cell chains spanned the entire SVZ (**Figures 4A, B**). In coronal sections of the control RMS the neuroblasts chains were organized in tight cell bundles (**Figure 4C**). In contrast, under radixin inhibition neuroblast chain formation in the SVZ were randomly oriented and displayed short intermittently interrupted cell chains (**Figure 4D**). The morphology of DCX^+^ cells wasless polarized and the neuroblastsformed clusters (**Figure 4E**). In the RMS, radixin inhibition resulted in similarly disorganized chains (**Figure 4F**). Previous studies show that the interaction between radixin and f-actin require phosphorylation of a threonine residue on the C-terminal end of radixin (Tsukita et al., 1997). After treatment with DX52-1, images indicate a reduced immunoreactivity for phosphorylated radixin in the RMS (**Figures 5A–D**).

**FIGURE 6:**
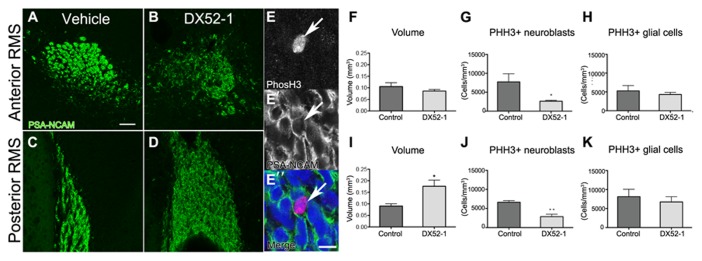
**Decreased neuroblast proliferation and accumulation of neuroblastsin the posterior RMS after radixin inhibition. (A, B)** PSA-NCAM immunoreactivity in the anterior RMS after 4 days of DX52-1 intracerebroventricular infusion of vehicle **(A)** or DX52-1 **(B)**. **(C, D)** PSA-NCAM immunoreactivity in the posterior RMS after 4 days of DX52-1 intracerebroventricular infusion of vehicle **(C)** or DX52-1 **(D)** shows an accumulation of PSA-NCAM^+^ neuroblasts in the posterior RMS after radixin inhibition. **(E, E′, E″)** Example of PHH3 **(E)** immunoreactive cell expressing PSA-NCAM **(E′**) and corresponding merged image in **(E″**). **(F)** There was no difference in the volume of the anterior RMS of vehicle and DX52-1 treated animals. **(I)** The volume of the posterior RMS was increased after infusion of DX52-1. **(G, J)** The number of PHH3 and PSA-NCAM double positive cells in the anterior **(G)** and posterior **(J)** RMS was decreased after 4 days of DX52-1 intracerebroventricular infusion. However, there was no difference in the number of PHH3 positive/PSA-NCAM negative cells in the anterior **(H)** or posterior RMS **(K)** (*p < 0.05, **p < 0.01). Scale bar in **(A)** = 50 μm, scale bar in (E″) = 10 μm.

The phenotype of neuroblasts described above is likely the cause of accumulation of neuroblasts in the anterior SVZ as evident by PSA-NCAM immunoreactive cells in coronal sections after DX52-1 infusions, in the posterior RMS (**Figures 6A–D**). In the posterior RMS the volume of the was increased (**Figure 6I**) whereas no difference was found in the volume of the anterior RMS (**Figure 6F**).

### RADIXIN INHIBITION AFFECTS PROLIFERATION OF NEUROBLASTS IN THE RMS

High amounts of radixin have been demonstrated in the cleavage furrow of dividing cells (Sato et al., 1991), suggesting an involvement of this protein in cell proliferation. A large proportion of neuroblasts divide en route from the SVZ to the OB (Luskin, 1993). Quantifying the number of cells expressing both the cell cycle marker PHH3 and PSA-NCAM (**Figures 6E, E′, E″**) revealed that fewer neuroblasts divided after radixin inhibition in both the anterior and posterior RMS (**Figures 6G, J**). However, there was no difference in proliferation in the glial population (PSA-NCAM negative cells) in the RMS (**Figures 6H, K**). To assess induction of apoptosis due to the DX52-1 treatment *in vivo* we analyzed the number of TUNEL positive cells in the RMS; however, no significant difference was detected (**Figure 2C**).

### ALTERED PROTEIN EXPRESSION AFTER RADIXIN INHIBITION

**FIGURE 7:**
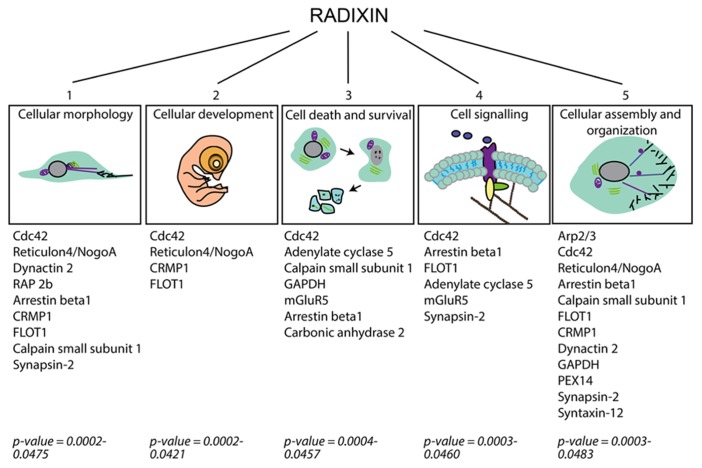
**Schematic figure categorizing cellular functions affected in the SVZ under radixin inhibition according to IPA analysis of the altered proteins as detected by proteome analysis.** Proteins allocated to each cellular function group are presented and the associated *p*-value for their enrichment in the group of altered proteins.

To study protein expression changes after treatment with DX52-1, the ipsilateral SVZ was dissected after 4 days of intracerebroventricular infusion of DX52-1 or vehicle (each *n* = 3). Using isobaric labeling and LC-MS/MS, 32 proteins were identified with significantly changed expression levels after treatment (**Table 1**). Functional analysis of all significantly changed proteins was performed using IPA (Ingenuity Systems, Redwood City, CA, USA) identifying two associated functional networks: (1) cell morphology, cellular development, small molecule biochemistry (enrichment score = 48); (2) cell-to-cell signaling and interaction, cellular development, developmental disorder (enrichment score = 30). The protein list was enriched for proteins involved in a number of basic molecular and cellular functions including cellular assembly and organization, which correlates well with the predicted functions of radixin (**Figure 7**).

**Table 1:** Proteins with altered expression level after radixin inhibition in the SVZ as detected by isobaric labeling and mass spectrometry.

Accession no.	Description	Foldchange	*p*-value	CV (%)
Q9WV97	Mitochondrial import inner membrane translocase subunit Tim9	1.21	0.05	8.13
P61227	Ras-related protein Rap-2b	1.20	0.05	5.73
Q6AYH5	Dynactin subunit 2	1.19	0.02	5.45
P31662	Orphan sodium- and chloride-dependentneurotransmitter transporter NTT4 (Slc6a17)	1.13	0.05	4.58
G3V7P1	Syntaxin-12	1.13	0.03	1.65
Q63537	Synapsin-2	1.12	0.02	3.91
Q6P9V9	Tubulin alpha-1B chain	1.12	0.02	3.60
Q6P7B0	Tryptophanyl-tRNA synthetase, cytoplasmic	1.12	0.01	1.65
P50408	V-type proton ATPase subunit F	1.11	0.00	1.91
Q9JK11	Reticulon-4/Nogo A	1.11	0.03	2.31
P13221	Aspartate aminotransferase, cytoplasmic	1.10	0.02	3.11
P21571	ATP synthase-coupling factor 6, mitochondrial	1.10	0.02	2.76
P04797	Glyceraldehyde-3-phosphate dehydrogenase (GAPDH)	1.10	0.01	2.37
O35824	DnaJ homolog subfamily A member 2	1.09	0.05	3.08
P04636	Malatedehydrogenase, mitochondrial	1.08	0.02	2.64
Q4KM73	UMP-CMP kinase	1.08	0.03	1.81
Q8CFN2	Cell division control protein 42 (Cdc42)	1.08	0.01	0.98
Q63569	26S protease regulatory subunit 6A	1.04	0.02	0.90
P27139	Carbonic anhydrase 2	0.97	0.00	0.59
Q62950	Dihydropyrimidinase-related protein 1	0.95	0.04	1.85
Q99PD4	Actin-related protein 2/3 complex subunit 1A	0.94	0.01	2.03
Q568Z9	Phytanoyl-CoA hydroxylase-interacting protein	0.91	0.02	3.26
O70196	Prolyl endopeptidase	0.90	0.01	2.25
Q04400	Adenylate cyclase type 5	0.89	0.02	3.34
Q9ESB5	N-terminal EF-hand calcium-binding protein 1 (Necab1)	0.87	0.03	3.62
Q642G4	Peroxisomal membrane protein PEX14	0.86	0.02	4.39
Q9Z1E1	Flotillin-1/Reggie 2	0.85	0.03	4.19
B0BN85	Suppressor of G2 allele of SKP1 homolog	0.85	0.01	3.49
P29066	Beta-arrestin-1	0.84	0.01	3.56
P31424	Metabotropic glutamate receptor 5	0.82	0.01	5.03
Q64537	Calpain small subunit 1	0.80	0.04	9.91
Q9JJ54	Heterogeneous nuclear ribonucleo protein D0	0.80	0.04	9.27

*Coefficient of variance, CV; *n *= 3, (*p* < 0.05).*

A number of proteins involved in cell-to-cell signaling and interaction changed expression levels, such as the metabotropic glutamate receptor 5 (mGluR5), arrestinβ1 and adenylatecyclase 5. The cytoskeleton components dynactin and alpha tubulin were enriched after DX52-1 treatment. Furthermore, proteins involved in molecular and vesicular transport, such as mitochondrial import inner membrane translocase, Scl6a17, syntaxin-12, synapsin-2, peroxisomal membrane protein PEX14 and flotillin-1 were altered. The suppressor of G2 allele SKP1 homolog, a protein regulating the transition from G2 to M-phase, was reduced, corroborating the *in vivo* results of decreased proliferation after radixin inhibition.

Altered proteins involved in protein metabolism include cytoplasmic tryptophanyl-tRNAsynthetase, V-type proton ATPase, cytoplasmic aspartate aminotransferase, mitochondrial ATP synthase-coupling factor 6, and heterogeneous nuclear riboprotein D0.

## DISCUSSION

The data presented in the current study suggest a role for radixin in neuronal progenitor migration and proliferation. We have previously shown that radixin is specifically expressed in migrating neuroblasts in the RMS of the adult rodent brain (Persson et al., 2010). The current study confirms the expression of radixin in PSA-NCAM^+^ migratory cells, both *in vivo* and *in vitro*. Blocking radixin with the quinocarmycin analog DX52-1 *in vitro* resulted in a dose-dependent downregulation of neuroblast migration from SVZ explants. Under control conditions similar numbers of PSA-NCAM^+^ neuroblasts and Sox2^high^ expressing glial cells migrate from the explants. The motility of glial cells was not affected by the inhibitor, supporting previous results describing the expression of ezrin, but not radixin, in glial cells (Cleary et al., 2006; Persson et al., 2010).

DX52-1 specifically binds radixin at low concentration and inhibits its binding to f-actin and the transmembrane protein CD44 (Kahsai et al., 2006). At concentrations above those used in our study, DX52-1 are reported to interact with additional proteins, such as the other ERM proteins, ezrin and moesin, and galectin-3 (Kahsai et al., 2006). Treatment with a low dose of DX52-1 in Madin-Darby canine kidney (MDCK) epithelial cell cultures revealed a decreased ability for wound closure after radixin inhibition (Kahsai et al., 2006), suggesting a role for radixin in migration and/or proliferation of epithelial cells. The selective inhibition of neuroblasts in our study confirms the specificity of DX52-1 for radixin, since Sox2^high^ glial cells remained migratory in the migration assay. In addition, continued migration of glial cells indicates that the inhibitor is not generally toxic to cells. Furthermore, we can exclude DX52-1 toxicity in the neurogenic niche since the rate of apoptosis or cell death was not increased, neither *in vitro* nor *in vivo*.

Intracerebroventricular infusion of DX52-1 resulted in distortion of neuroblast chain formation in the SVZ and the RMS. The accumulation of neuroblasts in the posterior parts of the RMS suggests that fewer neuroblasts migrate through the RMS. This was corroborated by an increased volume in the posterior RMS. However, this accumulation was not sufficient to cause any significant decrease in the volume of the anterior RMS indicating that neuroblast migration in the SVZ and posterior RMS may be hampered although sufficient to proceed through the RMS. A longer infusion period than 4 days may be required to reveal an effect along the entire RMS. Phosphorylation of radixin enables its binding to the actin cytoskeleton under control conditions. After DX52-1 treatment *in vivo, *thelevel of phosphorylated radixin immunoreactivity was low in the RMS. Thus, the aberrant neuroblast migration could be a result of decreased phosphorylation of radixin.

Furthermore, radixin has been shown to concentrate in the cleavage furrow of dividing cells (Sato et al., 1991) and may have a role in proliferation. We demonstrate a selective decrease in neuroblast proliferation in the RMS after intracerebroventricular infusion of DX52-1. Proliferation of other cell types (PSA-NCAM negative) was not affected. These data are in accordance with a study that tested DX52-1as a chemotherapeutic agent after a screening for molecules affecting growth of melanoma cells, which also express radixin (Plowman et al., 1995).

We have determined the effects on neuroblast migration and proliferation *in vivo *after DX52-1 infusion. However, the compound may have additional effects which are not related to neuroblast migration and proliferation. We could discern a moderate morphology change in the microglia population and an increase in the immunoreactivity of GFAP in the SVZ and corpus callosum (data not shown). Microdissection of DX52-1 treated brains indicated affected areas outside the SVZ/RMS and we observed vasculature changes in the thalamus, occasionally ventricle enlargement and softening of white matter tissue (data not shown). This may be explained by altered functions in non-neuroblast radixin expressing cells.

Considering recent evidence for regulation of cell functions by radixin other than migration (Loebrich et al., 2006; Tang et al., 2007; Valderrama et al., 2012), we quantified protein changes in the SVZ using a proteomic approach to identify biological functions affected by radixin inhibition in the neurogenic niche. It is important to consider that the proteomics analysis was based on a material of mixed cell types, and includes both intra- and extra-cellular proteins. This approach enables detection of general protein changes, including both primary and secondary events to the treatment. Our results show that the majority of the altered proteins have a role in cellular morphology and cellular assembly and organization which match our *in vitro* and *in vivo* results of radixin inhibition. Of the altered proteins 31% are abundantly expressed in the RMS according to the Allen brain atlas (http://mouse.brain-map.orghttp://mouse.brain-map.org), for example Reticulon 4/Nogo-A, Dynactin 2, and suppressor of G2 allele of SKP homolog. Cdc42 and Rac1 are members of the Rho family of small GTP-binding proteins, and radixinis known to interact with several Rho GTP-binding proteins and to regulate Rac1 activity (Takahashi et al., 1998; Hamada et al., 2001; Valderrama et al., 2012). Both Cdc42 and Rac1 are involved in neuronal embryonic migration but may have different roles (Konno et al., 2005). Cdc42 is for instance important for the guiding cues of the Slit-Robo pathway (Wong et al., 2001), suggesting a specific role in RMS migration. Calpain small subunit 1 is present in both calpain 1 and 2 and calpains regulate cell migration and adhesion (Huttenlocher et al., 1997; Dourdin et al., 2001). Recently, calpain 1 expression was shown to be high in neural stem cells and decreased during differentiation and calpain 1 inhibition increased neural stem cell differentiation (Santos et al., 2012). Contrary, calpain 2 was increased during neural stem cell differentiation (Santos et al., 2012). Furthermore, arrestins were initially acknowledged for their role in receptor internalization; however, recent evidence suggest a role for β-Arrestin-2 in promoting actin polymerization and migration of leukocytes (Zoudilova et al., 2010). The interaction of radixin with proteins identified in the proteomics analysis may be direct or indirect, or induced as a compensatory action to radixin inhibition. Future studies will have to address these issues. Our data suggest that radixin likely interacts with several different signaling or scaffolding proteins to mediate and/or regulate dynamic actin rearrangement.

The effects of acute radixin inhibition in this study suggest that the functions of radixin in neuroblasts are not compensated by other actin binding proteins described in RMS neuroblasts, such as Girdin (Wang et al., 2011). However, the antibody against phosphorylated Ezrin/Radixin/Moesin does not discriminate between the three ERM proteins and thus, changes in phosphorylation levels could be due to any of the three proteins. We can exclude ezrin from being affected, since we do not see any effect on migration of glial cells, which express high levels of ezrin (Gronholm et al., 2005; Cleary et al., 2006; Persson et al., 2010) even at high doses of DX52-1. This confirms that DX52-1 seems not to generally block a common site on all three ERM proteins. In Persson et al. (2010), we show that close to all neuroblasts express radixin as the main ERM protein but also to a lower degree moesin, leaving the possibility that radixin and moesin could be affected by DX52-1. In the current study we observe that all radixin positive cells in the RMS loose their pERM immunoreactivity upon DX52-1 incubation. We conclude from this that radixin is dephosphorylated and inactivated by DX52-1. However, we cannot exclude an additional contribution to the cellular effects from a possible inactivation of moesin.

## Conflict of Interest Statement

The authors declare that the research was conducted in the absence of any commercial or financial relationships that could be construed as a potential conflict of interest.
